# Comparison of the Expression Changes after Botulinum Toxin Type A and Minocycline Administration in Lipopolysaccharide-Stimulated Rat Microglial and Astroglial Cultures

**DOI:** 10.3389/fcimb.2017.00141

**Published:** 2017-04-26

**Authors:** Anna Piotrowska, Katarzyna Popiolek-Barczyk, Flaminia Pavone, Joanna Mika

**Affiliations:** ^1^Department of Pain Pharmacology, Institute of Pharmacology, Polish Academy of SciencesKrakow, Poland; ^2^CNR, Institute of Cell Biology and NeurobiologyRome, Italy; ^3^IRCCS, Santa Lucia FoundationRome, Italy

**Keywords:** BoNT/A, inflammation, inflammatory factors, intracellular pathways, TLR2

## Abstract

Botulinum neurotoxin type A (BoNT/A) and minocycline are potent drugs used in clinical therapies. The primary molecular mechanism of BoNT/A is the cleavage of SNARE proteins, which prevents cells from releasing neurotransmitters from vesicles, while the effects of minocycline are related to the inhibition of p38 activation. Both BoNT/A and minocycline exhibit analgesic effects, however, their direct impact on glial cells is not fully known. Therefore, the aim of the present study was to determine the effects of those drugs on microglial and astroglial activity after lipopolysaccharide (LPS) stimulation and their potential synergistic action. Our results show that BoNT/A and minocycline influenced primary microglial cells by inhibiting intracellular signaling pathways, such as p38, ERK1/2, NF-κB, and the release of pro-inflammatory factors, including IL-1β, IL-18, IL-6, and NOS2. We have revealed that, in contrast to minocycline, BoNT/A treatment did not decrease LPS-induced release of pro-inflammatory factors in the astroglia. In addition, BoNT/A decreased SNAP-23 in both types of glial cells and also SNAP-25 expressed only in astrocytes. Moreover, BoNT/A increased TLR2 and its adaptor protein MyD88, but not TLR4 exclusively in microglial cells. Furthermore, we have shown the impact of BoNT/A on microglial and astroglial cells, with a particular emphasis on its molecular target, TLR2. In contrast, minocycline did not affect any of those factors. We have revealed that despite of different molecular targets, minocycline, and BoNT/A reduced the release of microglia-derived pro-inflammatory factors. In conclusion, we have shown that BoNT/A and minocycline are effective drugs for the management of neuroinflammation by dampening the activation of microglial cells, with minocycline also affecting astroglial activity.

## Introduction

Botulinum toxin A (BoNT/A) is a neurotoxin produced by the anaerobic bacteria *Clostridium botulinum*. BoNT/A interferes with neuronal transmission by blocking the release of neurotransmitters (Pantano and Montecucco, 2014). The molecular action of this neurotoxin is the Zn^2+^-endopeptidase activity-mediated cleavage of specific proteins involved in neuroexocytosis, such as synaptosomal-associated proteins (SNAPs) (Schiavo et al., 2000; Luvisetto et al., 2003, 2006; Montecucco and Molgó, 2005; Snyder et al., 2006). Presently, BoNT/A plays a significant role in the management of a wide range of medical conditions. Recent studies, including our own, have been devoted to the use of BoNT/A in pain therapy (Jabbari, 2008; Ashkenazi, 2010; Mika et al., 2011; Francisco et al., 2012; Vacca et al., 2013; Zychowska et al., 2016). Previous research had shown that BoNT/A strongly inhibits the release of neurotransmitters and neuropeptides, such as glutamate (Cui et al., 2004), substance P (Welch et al., 2000), and calcitonin gene-related peptide (CGRP) (Durham et al., 2004; Meng et al., 2007), which results from the ability of this toxin to cleave SNAP-25, one of the crucial SNARE proteins involved in neuroexocytosis (Schiavo et al., 1993; Rossetto et al., 2001; Montecucco et al., 2004).

In 2011, we showed that BoNT/A not only attenuated neuropathic pain-related behaviors in rats by impeding injury-activated neuronal function, but also reduced the activation of spinal microglia (Mika et al., 2011). In CCI-exposed mice, BoNT/A reduced the following: the number of astrocytes, the percentage of active astrocytes, and the activation of microglia in neuropathic animals after chronic morphine treatment (Vacca et al., 2013). Recently, we have shown that BoNT/A restores the neuroimmune balance between pronociceptive (IL-1β and IL-18) and antinociceptive (IL-10 and IL-1RA) factors within the spinal cord of neuropathic rats (Zychowska et al., 2016). Recent reports suggest that BoNT/A modulates the immune response through a TLR2-dependent pathway in macrophages (Kim et al., 2015). However, the exact modulation of this pathway by BoNT/A has not been explored.

Reduction of glial cell activation, especially microglia, and the associated neuroinflammation was suggested as an effective neuropathic pain treatment. Many reports have noted that microglial inhibitors, such as minocycline, prevent the development of neuropathy in animal models (Sweitzer et al., 2001; Tikka et al., 2001; Mika et al., 2007, 2009, 2010, 2014; Cui et al., 2008; Rojewska et al., 2014). Minocycline, a semisynthetic tetracycline antibiotic, which acts against both Gram-positive and Gram-negative bacteria, diminished neuropathic pain by reducing microglial cell activation and attenuated the expression of numerous pronociceptive factors (Popiolek-Barczyk et al., 2014a; Rojewska et al., 2014). Our latest data have revealed that minocycline slightly enhances the analgesic effects of BoNT/A in CCI-exposed animals (Zychowska et al., 2016).

Basing on previous studies, we have suggested that the analgesic action of BoNT/A might be related to the modulation of glial cell activity and/or gene expression. Therefore, in the present study, we explored possible effects of BoNT/A on microglia and astroglia in an *in vitro* model of LPS-induced glial cell activation and compared its effectiveness with minocycline. We examined the influence of BoNT/A and minocycline on microglial and astroglial cell viability. Using qRT-PCR and Western blot techniques, we explored the influence of BoNT/A and minocycline on SNAP-23 and -25, as well as immune factors (MMP9, NOS2, IL-1β, IL-18, IL-6, IL-10, IL-1RA, IL-18BP). We also analyzed the protein levels of related intracellular signaling pathways (NF-κB, p38 MAPK, and ERK1/2) which underlie the development of neuroinflammation. We also examined the effects of both compounds on the mRNA and protein levels of TLR2 and TLR4. Additionally, we assessed whether the administration of BoNT/A and minocycline could be associated with any additive effects.

## Materials and methods

### Microglial and astroglial cell cultures

Neonatal models of primary cultures of microglial and astroglial cells were used in our *in vitro* studies as had been shown previously (Popiolek-Barczyk et al., 2014a, 2015; Piotrowska et al., 2016; Rojewska et al., 2016). Both types of cell cultures were prepared from 1-day-old Wistar rats according to the procedure described by Zawadzka and Kaminska (2005). The cells were isolated from the cerebral cortex and placed in poly-l-lysine-coated, 75-cm^2^ culture bottles at a density of 3 × 10^5^ cells/cm^2^ in high-glucose DMEM with GlutaMAX (Gibco, New York, USA), heat-inactivated 10% fetal bovine serum, 0.1 mg/ml streptomycin, and 100 U/ml penicillin (Gibco, New York, USA). The cultures were maintained at 37°C in 5% CO_2_. On the fourth day, the culture medium was changed. On the ninth day, the cultures were gently shaken and centrifuged to recover any loosely adherent microglia. Then, the medium was changed, and on the twelfth day the microglia were recovered again. Once more, the culture medium was replaced, and the cultures were allowed to grow on a rotary shaker at 37°C for 24 h (200 rpm) to remove the remaining non-adherent cells. The medium was removed, and astrocytes were cultured on plates for 3 days. Then, the astrocytes were trypsinized (0.005% trypsin EDTA solution, Sigma-Aldrich, St. Louis, USA). Microglia/astrocytes were seeded at a final density of 1.2 × 10^6^ cells per 6-well plate for protein analysis and 4 × 10^4^ cells per 96-well plates for MTT analysis in the culture medium, and then, they were incubated for 48 h. Primary microglial and astrocyte cell cultures were treated with BoNT/A [0.01, 0.1, 1, 5, 50, 100 nM] and/or minocycline [MC; 20 μM] 30 min before LPS (lipopolysaccharide from *Escherichia coli* 0111:B4; Sigma-Aldrich, St. Louis, USA) administration [100 ng/mL] LPS dose was selected basing on the literature (Zawadzka and Kaminska, 2005; Przanowski et al., 2014, and our own experiences Rojewska et al., 2014, 2016; Malek et al., 2015; Popiolek-Barczyk et al., 2015; Piotrowska et al., 2016) and incubated for 1 h (for the analysis of intracellular pathway activation) and 24 h (for the analysis of gene expression, MTT, Popiolek-Barczyk et al., 2015; Piotrowska et al., 2016; Rojewska et al., 2016). The purity of LPS used in our study and its specificity for TLR4 was validated by Douville et al. (2010). The authors revealed that pretreatment with TLR4 blocking antibodies abrogate the capacity of LPS to stimulate cytokine production. The BoNT/A concentration was selected basing on cell viability, which was similar to the dose used in Kim and colleagues' study applied in macrophages. The concentration of minocycline was selected basing on previous studies (Piotrowska et al., 2016; Rojewska et al., 2016). To identify microglia and astrocytes in the cell cultures, we used immunostaining for IBA1 (a microglial marker, SC-327 225, Santa Cruz Biotechnology Inc., Santa Cruz, USA) and GFAP (an astrocyte marker, SC-166 458, Santa Cruz Biotechnology Inc., Santa Cruz, USA). We obtained highly homogeneous microglial and astroglial populations (more than 95% positive for IBA1 and GFAP, respectively, Zawadzka and Kaminska, 2005). Only the minimal essential number of animals was used, and all of the procedures were performed according to the recommendations of IASP (Zimmermann, 1983) and the NIH Guide for the Care and Use of Laboratory Animals. The study was carried out in accordance with the recommendations of local Ethics Committee (Krakow, Poland), permission number: 1055.

### Drug administration

Minocycline hydrochloride (MC) was obtained from Sigma (USA), and the BoNT/A was a kind gift from Prof. C. Montecucco (Department of Experimental Biomedical Sciences, University of Padova, Italy). For the treatments, MC and BoNT/A were dissolved in water, and the control groups received vehicle (water).

### Biochemical test

#### Cell viability assay

The cell viability after the BoNT/A and minocycline treatments alone and after the LPS administration was determined by a tetrazolium salt 3-[4,5-dimethylthiazol-2-yl]-2,5-diphenyltetrazolium bromide assay (MTT, Sigma-Aldrich, Germany). After 24 h of treatment with different concentrations of BoNT/A [0.01, 0.1, 1, 5, 10, 50, 100 nM] and minocycline [20 μM] with or without LPS [100 ng/mL], MTT (at a concentration of 0.15 mg/mL) was added to each well, and the cells were incubated for 2 h at 37°C. Next, the culture medium was discarded, and 0.1 M HCl in isopropanol was added to dissolve the formazan dye. The absorbance values were measured using a Multiskan Spectrum apparatus at 570 nm. The data were normalized to the absorbance in the control group (vehicle-treated non-stimulated cells) and expressed as a percentage of the mean of control ± SEM.

#### Analysis of protein levels (western blot)

The cell lysates (in RIPA buffer with a protease inhibitor cocktail) from primary microglial and astroglial cultures for Western blot analysis were collected 1 or 24 h after LPS stimulation. Then, the reaction mixtures were cleared by centrifugation (14,000 × g for 30 min at 4°C). Samples containing 20 μg of protein were heated in a loading buffer (4x Laemmli Buffer, Bio-Rad, Warsaw, Poland) for 5 min at 98°C. Then, all samples were resolved on 4–15% Criterion™ TGX™ precast polyacrylamide gels (Bio-Rad, Warsaw, Poland). The proteins were transferred to Immune-Blot PVDF membranes (Bio-Rad, Warsaw, Poland) with a semi-dry transfer (30 min, 25 V). The membranes were blocked for 1 h at RT using 5% non-fat dry milk (Bio-Rad) in Tris-buffered saline with 0.1% Tween-20 (TBST). The membranes were then washed in TBST and incubated overnight at 4°C with the following primary antibodies: rabbit polyclonal IL-1β (Abcam) 1:1,000, IL-18 (R&D Systems) 1:1,000, IL-6 (Invitrogen) 1:500, NOS2 (Santa Cruz) 1:500, IL-1RA (Abcam) 1:1,000, IL-18BP (Novus Biologicals) 1:1,000, IL-10 (Invitrogen) 1:500, p38 MAPK (Cell Signaling) 1:1,000, p-p38 MAPK (Cell Signaling) 1:1,000, ERK1/2 (Cell Signaling) 1:1,000, p-ERK1/2 (Cell Signaling) 1:1,000, NF-κB (Santa Cruz) 1:500, p-NF-κB (Santa Cruz) 1:500, SNAP-23 (ProteinTech) 1:1,000, SNAP-25 (ProteinTech) 1:1,000, TLR2 (Abcam) 1:500, MyD88 (Novus) 1:500, mouse polyclonal TLR4 (Santa Cruz) 1:500, and GAPDH (Millipore) 1:5,000. The membranes were then incubated for 1 h in horseradish peroxidase-conjugated anti-rabbit or anti-mouse secondary antibodies at a dilution of 1:5,000. Solutions from the SignalBoost™ Immunoreaction Enhancer Kit (Merck Millipore Darmstadt, Germany) were used to dilute the primary and secondary antibodies. The membranes were washed 2 times for 2 min each, and 3 times for 5 min each with TBST. The Clarity™ Western ECL Substrate (Bio-Rad, Warsaw, Poland) was used to detect immunocomplexes, which were then visualized using a Fujifilm LAS-4000 FluorImager system. The Fujifilm Multi Gauge software was used to quantify the relative levels of immunoreactivity.

### Statistical analyses

**Figure 1:**
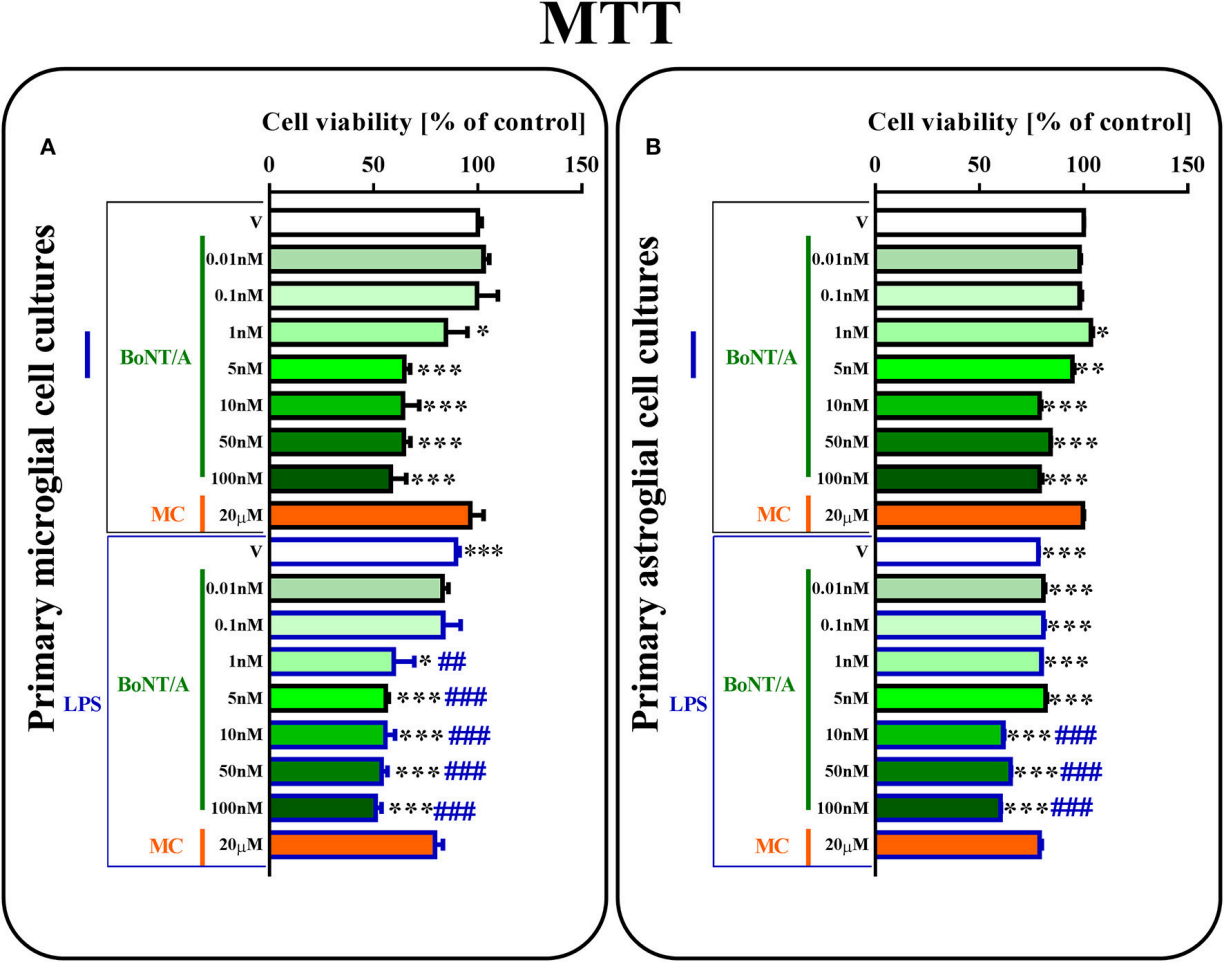
**The influence of BoNT/A and minocycline (MC) on cell viability in vehicle- and LPS-treated primary microglial (A)** and astroglial **(B)** cell cultures. BoNT/A [0.01, 0.1, 1, 5, 10, 50, 100 nM] and minocycline [20 μM] were added to the culture medium 30 min before LPS [100 ng/mL] treatment, and then the cells were cultured for 24 h. The results are presented as a percentage of control (vehicle-treated non-stimulated cells) as the mean ± SEM of 3–4 independent experiments. The results were evaluated using one-way analysis of variance (ANOVA) followed by Bonferroni's *post hoc* test to assess differences between the treatment groups. Significant differences in comparison with the control group (vehicle-treated non-stimulated cells) are indicated by ^*^P < 0.05, ^**^P < 0.01, ^***^P < 0.001; differences between LPS-treated and BoNT/A- or MC-treated cells are indicated by ^##^*P* < 0.01, ^###^*P* < 0.001.

The results of the cell viability and Griess assays (Figure 1) are presented as a percentage of the control (vehicle-treated cells) as the mean ± SEM of 3–4 independent experiments. The results of the qRT-PCR (Supplementary Materials) and Western blot (**Figures 3–6**) are presented as the fold change compared with the control group (vehicle-treated cells) as the mean ± SEM of 3–5 independent experiments. The results were evaluated using one-way analysis of variance (ANOVA) with Bonferroni's *post hoc* test to assess the differences between the treatment groups. All graphs and analyses were prepared using GraphPad Prism version 5.

## Results

### The influence of BoNT/A and minocycline on cell viability in vehicle- and LPS-treated microglial and astroglial cells

Using primary microglial and astroglial cell cultures, we examined the effects of different doses of BoNT/A [0.01, 0.1, 1, 5, 10, 50, 100 nM] and minocycline [20 μM] on cell viability. LPS treatment [100 ng/mL] resulted in lower cell viability in primary microglial and astroglial cultures compared to vehicle-treated non-stimulated cells (Figures 1A,B). As shown in Figure 1, 24-h BoNT/A-treatment at the doses of 1, 5, 10, 50, 100 nM decreased microglial cell viability, but BoNT/A-treatment at the doses of 0.01, 0.1 nM and MC-treatment at the dose of 20 μM did not change microglial cell viability, as measured by the MTT reduction assay (Figure 1A). In astroglial cell cultures, 24-h BoNT/A-treatment at the doses of 5, 10, 50, 100 nM decreased cell viability, but BoNT/A-treatment at the doses of 0.01, 0.1, 1 nM and MC-treatment at the dose of 20 μM did not change cell viability, as measured by the MTT reduction assay. Basing on the results obtained from the aforementioned studies and the literature data, we decided to perform subsequent experiments using BoNT/A at the dose of 0.1 nM and minocycline at the dose of 20 μM.

### The influence of BoNT/A and minocycline on SNARE proteins in vehicle- and LPS-treated microglial and astroglial cells

**Figure 2:**
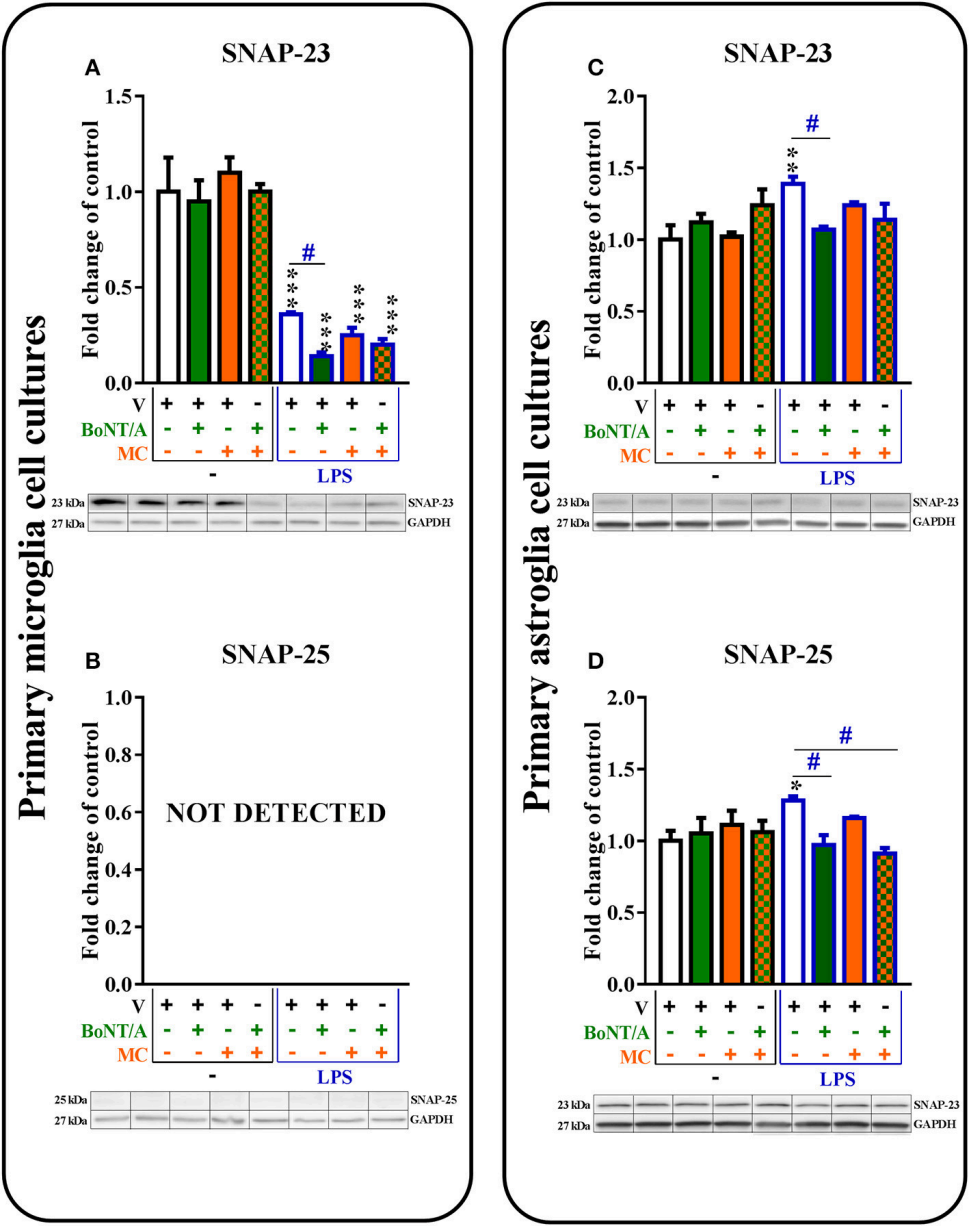
**The influence of BoNT/A and/or minocycline (MC) on SNAP-23 (A,C)** and SNAP-25 **(B,D)** protein levels in vehicle- and LPS-treated primary microglial **(A,B)** and astroglial **(C,D)** cell cultures. Microglial and astroglial cells were treated with BoNT/A [0.1 nM] and/or minocycline [20 μM] for 30 min and then with LPS [100 ng/mL] for 24 h **(A–D)**. The representative bands are shown below each column of the respective group on the graph and come from the same membrane photo. Samples from different groups were not next to each other so were cut from different locations and set together. The data are presented as the fold change compared with the control group (vehicle-treated non-stimulated cells) as the mean ± SEM of 3–5 independent experiments. The results were evaluated using one-way analysis of variance (ANOVA) followed by Bonferroni's *post hoc* test to assess differences between the treatment groups. Significant differences in comparison with the control group (vehicle-treated non-stimulated cells) are indicated by ^*^P < 0.05, ^**^P < 0.01, ^***^P < 0.001; differences between LPS-treated and BoNT/A- and/or MC-treated cells are indicated by ^#^*P* < 0.05.

*SNAP-23* mRNA (please see Supplementary Material) was decreased in microglia and increased in astroglia after LPS stimulation. Additionally, SNAP-23 protein was decreased in microglia (1.0 ± 0.08 vs. 0.36 ± 0.01) and increased in astroglia (1.0 ± 0.1 vs. 1.39 ± 0.05) after LPS treatment compared with vehicle-treated non-stimulated cells (control group). BoNT/A diminished the protein levels of SNAP-23 from 0.36 ± 0.01 to 0.14 ± 0.0 in the microglia (Figure 2A) and from 1.39 ± 0.05 to 1.07 ± 0.02 in the astroglia (Figure 2C) in LPS-stimulated cells compared with that of vehicle-treated LPS-stimulated cells. Minocycline and combination treatments did not exert any influence on the protein levels of SNAP-23 in the microglial and astroglial cell cultures (Figures 2A,C).

*SNAP-25* mRNA (Table 1A in Supplementary Materials) and protein (Figure 2B) levels were not detected in microglia (in non-stimulated or LPS-treated). However, the presence of both *SNAP-25* mRNA (please see Supplementary Material) and protein (Figure 2D) were observed in astroglia. BoNT/A diminished LPS-induced increases in the protein levels of SNAP-25 in astroglia from 1.28 ± 0.0 to 0.91 ± 0.07 compared with vehicle-treated non-stimulated cells (Figure 2D). We obtained similar results in our mRNA studies (please see Supplementary Material). The combination treatments diminished the protein levels of SNAP-25 from 1.28 ± 0.0 to 0.91 ± 0.04 in the astroglia (Figure 2D) in LPS-stimulated cells compared with that of vehicle-treated LPS-stimulated cells. Minocycline did not influence the mRNA (please see Supplementary Material) or protein (Figure 2D) levels of SNAP-25 in the astroglial cell cultures.

### The influence of BoNT/A and minocycline on MMP9 and intracellular factors in vehicle- and LPS-treated microglial and astroglial cells

The mRNA levels of *MMP9* were strongly up-regulated after LPS stimulation, and those elevated levels were reduced by minocycline but not by BoNT/A in either glial cell types (please see Supplementary Material).

**Figure 3:**
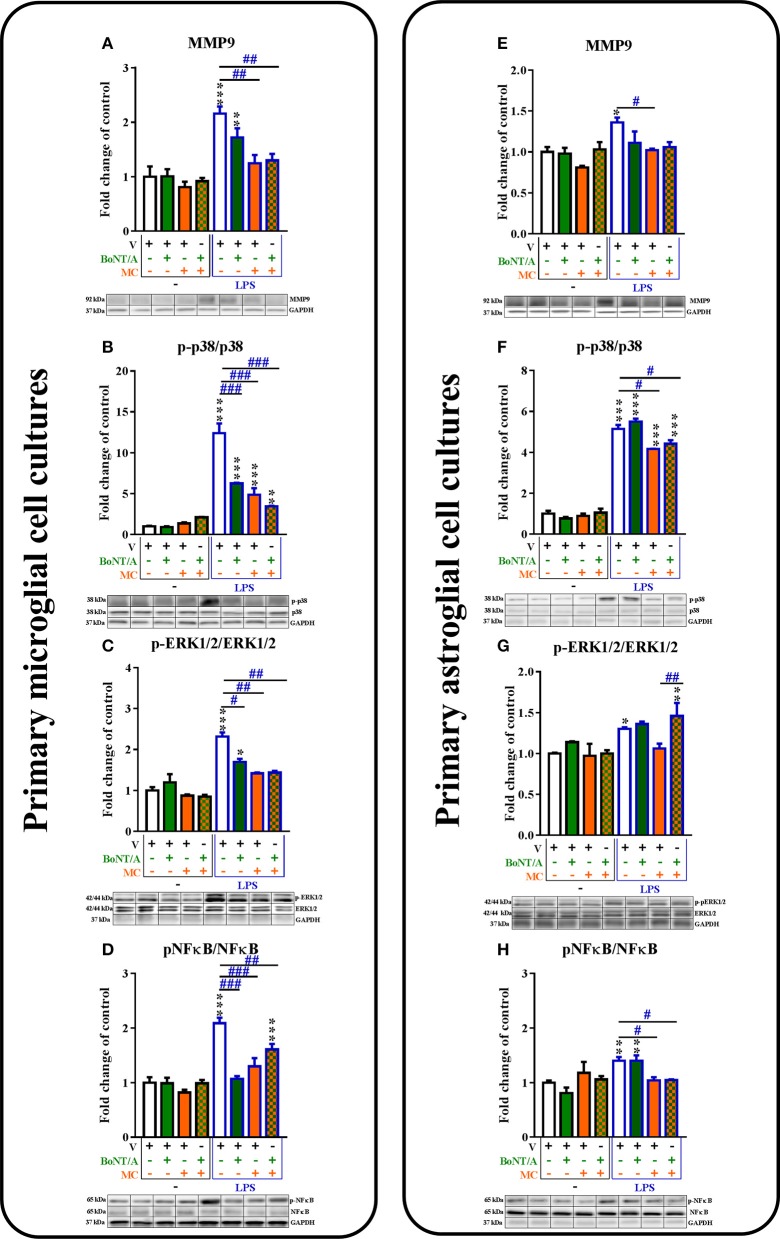
**The influence of BoNT/A and/or minocycline (MC) on MMP9 protein levels (A,E)** and p38 **(B,F)**, ERK1/2 **(C,G)** and NF-κB **(D,H)** phosphorylation in vehicle- and LPS-treated primary microglial **(A–D)** and astroglial **(E–H)** cell cultures. Microglial and astroglial cells were treated with BoNT/A [0.1 nM] and/or minocycline [20 μM] for 30 min and then with LPS [100 ng/mL] for 1 h **(B–H)** and 24 h **(A,E)**. The representative bands are shown below each column of the respective group on the graph and come from the same membrane photo. Samples from different groups were not next to each other so were cut from different locations and set together. The data are presented as the fold change compared with the control group (vehicle-treated non-stimulated cells) as the mean ± SEM of 3–5 independent experiments. The results were evaluated using one-way analysis of variance (ANOVA) followed by Bonferroni's *post hoc* test to assess differences between the treatment groups. Significant differences in comparison with the control group (vehicle-treated non-stimulated cells) are indicated by ^*^P < 0.05, ^**^P < 0.01, ^***^P < 0.001; differences between LPS-treated and BoNT/A- and/or MC-treated cells are indicated by ^#^*P* < 0.05, ^##^*P* < 0.01, ^###^*P* < 0.001.

The protein levels of MMP9 were significantly increased after LPS treatment compared to controls in both microglial and astroglial cell cultures (1.0 ± 0.2–2.16 ± 0.1; 1.0 ± 0.1–1.36 ± 0.1, respectively, Figures 3A,H). BoNT/A did not influence the protein levels of MMP9 in any of glial cultures (Figures 3A,H). Minocycline diminished the protein levels of MMP9 from 2.16 ± 0.1 to 1.25 ± 0.15 in the microglia (Figure 3A) and from 1.36 ± 0.1 to 0.9 ± 0.0 in the astroglia (Figure 3E) in LPS-stimulated cells compared with vehicle-treated LPS-stimulated cells. Combination treatments significantly decreased the protein levels of MMP9 from 1.3 ± 0.03 to 1.0 ± 0.0 in the microglia (Figure 3A) but not in the astroglia (Figure 3E) in LPS-stimulated cells compared with vehicle-treated LPS-stimulated cells.

**Figure 4:**
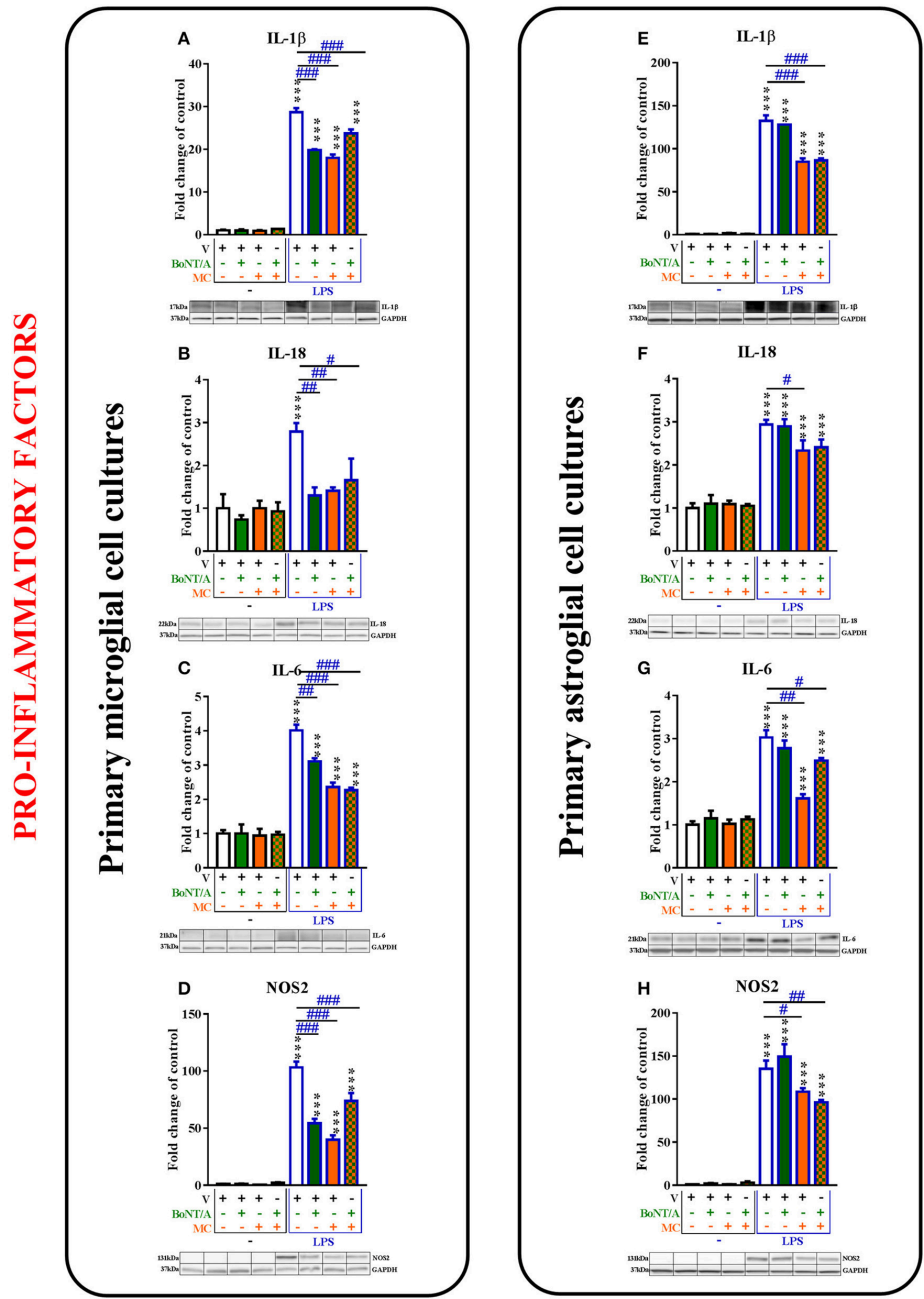
**The influence of BoNT/A and/or minocycline (MC) on IL-1β (A,E)**, IL-18 **(B,F)**, IL-6 **(C,G)**, and NOS2 **(D,H)** protein levels in vehicle- and LPS-treated primary microglial **(A–D)** and astroglial **(E–H)** cell cultures. Microglial and astroglial cells were treated with BoNT/A [0.1 nM] and/or minocycline [20 μM] for 30 min and then with LPS [100 ng/mL] for 24 h **(A–H)**. The representative bands are shown below each column of the respective group on the graph and come from the same membrane photo. Samples from different groups were not next to each other so were cut from different locations and set together. The data are presented as the fold change compared with the control group (vehicle-treated non-stimulated cells) as the mean ± SEM of 3–5 independent experiments. The results were evaluated using one-way analysis of variance (ANOVA) followed by Bonferroni's *post hoc* test to assess differences between the treatment groups. Significant differences in comparison with the control group (vehicle-treated non-stimulated cells) are indicated by ^***^P < 0.001; differences between LPS-treated and BoNT/A- and/or MC-treated cells are indicated by ^#^*P* < 0.05, ^##^*P* < 0.01, ^###^*P* < 0.001.

The protein levels of p-p38 MAPK were increased in the microglia from 1.0 ± 0.1 to 12.4 ± 1.2 (Figure 3B) and in the astroglia from 1.0 ± 0.1 to 5.2 ± 0.2 (Figure 3F) in LPS-stimulated cells compared with that of non-stimulated cells. BoNT/A decreased the phosphorylation of p38 in LPS-stimulated cells compared with that of vehicle-treated LPS-stimulated cells from 12.4 ± 1.2 to 6.26 ± 0.1 in microglia (Figure 3B) but not in astroglia (Figure 3F). Minocycline reduced the protein levels of p-p38 from 12.4 ± 1.2 to 4.87 ± 0.8 in the microglia (Figure 3B) and from 5.2 ± 0.2 to 4.17 ± 0.0 in the astroglia (Figure 4F) in LPS-stimulated cells compared with that of vehicle-treated LPS-stimulated cells. In addition, the combination treatments significantly decreased the protein levels of p-p38 from 12.4 ± 1.2 to 3.45 ± 0.1 in the microglia (Figure 3B) and from 5.2 ± 0.2 to 4.42 ± 0.2 in the astroglia (Figure 3F) compared with that of vehicle-treated LPS-stimulated cells.

The p-ERK1/2 protein levels were increased in the microglia from 1.0 ± 0.1 to 2.3 ± 0.1 (Figure 3C) and in the astroglia from 1.0 ± 0.1 to 1.3 ± 0.0 (Figure 3G) in LPS-stimulated cells compared with that of non-stimulated cells. BoNT/A decreased the phosphorylation of ERK1/2 in LPS-stimulated cells compared with that of vehicle-treated LPS-stimulated cells from 2.3 ± 0.1 to 1.7 ± 0.1 in microglia (Figure 3C) but not in astroglia (Figure 3G). Minocycline diminished the protein levels of p-ERK1/2 from 2.3 ± 0.1 to 1.4 ± 0.0 in the microglia (Figure 3C) but not in the astroglia (Figure 3G) in LPS-stimulated cells compared with that of vehicle-treated LPS-stimulated cells. In parallel, the combination treatments significantly decreased the protein levels of p-ERK1/2 from 2.3 ± 0.1 to 1.44 ± 0.0 in the microglia (Figure 3C) but not in the astroglia (Figure 3G) compared with that of vehicle-treated LPS-stimulated cells.

The p-NF-κB protein levels were increased in the microglia from 1.0 ± 0.1 to 2.1 ± 0.1 (Figure 3D) and in the astroglia from 1.0 ± 0.0 to 1.4 ± 0.1 (Figure 3H) in LPS-stimulated cells compared with that of non-stimulated cells. BoNT/A decreased the phosphorylation of NF-κB in LPS-stimulated cells compared with that of vehicle-treated LPS-stimulated cells from 2.1 ± 0.1 to 1.1 ± 0.1 in microglia (Figure 3D) but not in astroglia (Figure 3G). Minocycline diminished the protein levels of p-NF-κB from 2.1 ± 0.1 to 1.3 ± 0.2 in the microglia (Figure 3D) and from 1.4 ± 0.1 to 1.04 ± 0.1 in the astroglia (Figure 3H) in LPS-stimulated cells compared with that of vehicle-treated LPS-stimulated cells. Moreover, the combination treatments significantly decreased the protein levels of p-NF-κB from 2.1 ± 0.1 to 1.6 ± 0.1 in the microglia (Figure 3B) and from 1.4 ± 0.1 to 1.1 ± 0.0 in the astroglia (Figure 3F) in LPS-stimulated cells compared with that of vehicle-treated LPS-stimulated cells.

### The influence of BoNT/A and minocycline on pro-inflammatory factors in vehicle- and LPS-treated microglial and astroglial cells

The mRNA levels of pro-inflammatory factors (*IL-1*β*, IL-18, IL-6*, and *NOS2*) were significantly increased after LPS treatment compared to controls in the microglial and astroglial cell cultures. BoNT/A treatment decreased the mRNA levels of those factors in microglial but not in astroglial cell cultures. Minocycline decreased the mRNA levels of those factors in both cell cultures (please see Supplementary Material).

The IL-1β protein levels were significantly increased after LPS treatment compared to controls in microglial and astroglial cell cultures (from 1.0 ± 0.2 to 28.7 ± 1.0; from 1.0 ± 0.1 to 132.5 ± 6.4, respectively, Figures 4A,E). BoNT/A decreased the levels of IL-1β in LPS-stimulated cells compared with that of vehicle-treated LPS-stimulated cells from 28.7 ± 1.0 to 19.8 ± 0.2 in microglia (Figure 4A) but not in astroglia (Figure 4E). Minocycline diminished the protein levels of IL-1β from 28.7 ± 1.0 to 18.0 ± 0.8 in the microglia (Figure 4A) and from 132.5 ± 6.4 to 84.9 ± 4.0 in the astroglia (Figure 4E) in LPS-stimulated cells compared with that of vehicle-treated LPS-stimulated cells. In addition, the combination treatments significantly decreased the protein levels of IL-1β from 28.7 ± 1.0 to 23.7 ± 0.1 in the microglia (Figure 4B) and from 132.5 ± 6.4 to 86.5 ± 2.4 in the astroglia (Figure 4F) in LPS-stimulated cells compared with that of vehicle-treated LPS-stimulated cells.

**Figure 5:**
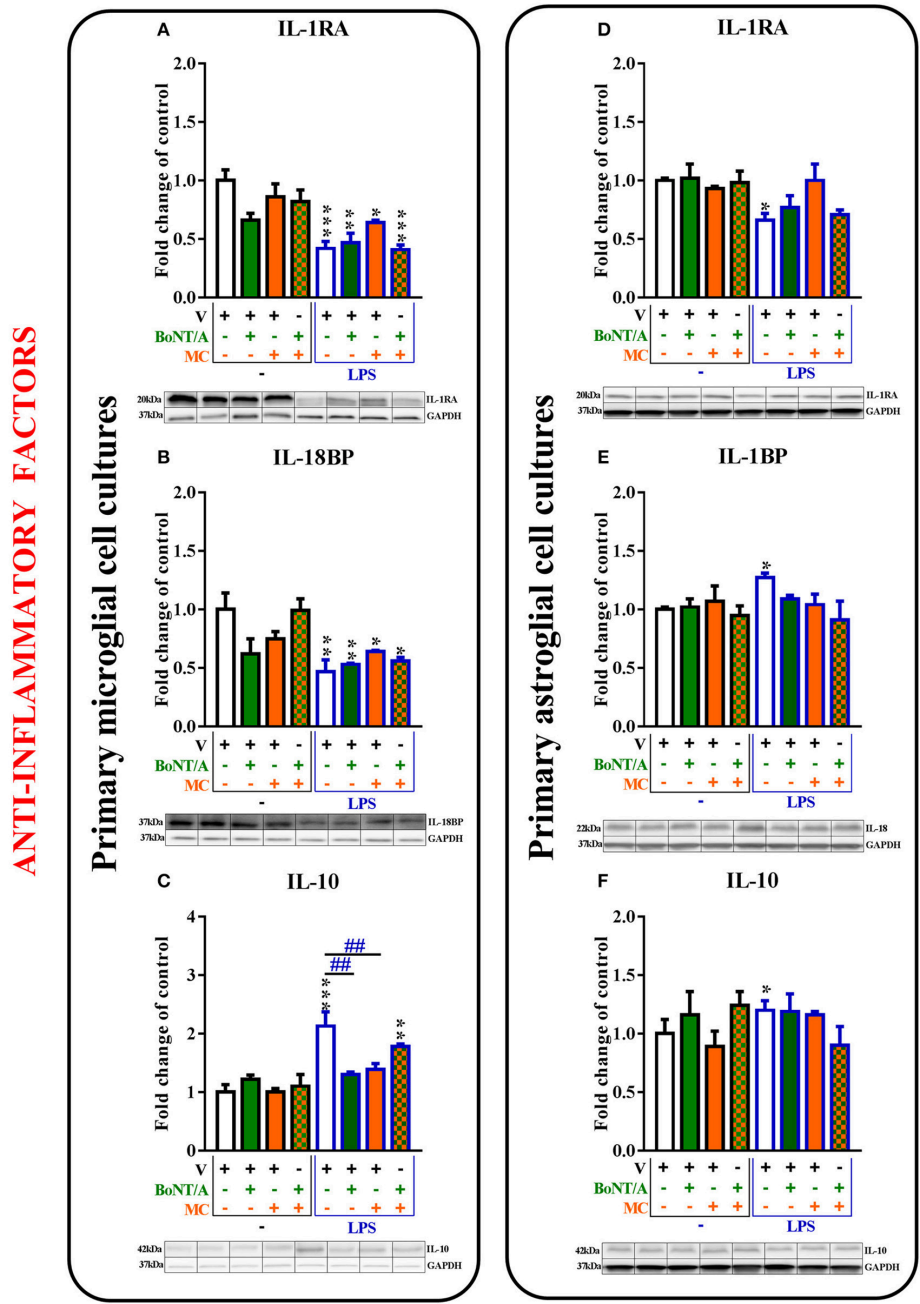
**The influence of BoNT/A and/or minocycline (MC) on IL-1RA (A,D)**, IL-18BP **(B,E)**, and IL-10 **(C,F)** protein levels in vehicle- and LPS-treated primary microglial **(A–D)** and astroglial **(E–H)** cell cultures. Microglial and astroglial cells were treated with BoNT/A [0.1 nM] and/or minocycline [20 μM] for 30 min and then with LPS [100 ng/mL] for 24 h **(A–H)**. The representative bands are shown below each column of the respective group on the graph and come from the same membrane photo. Samples from different groups were not next to each other so were cut from different locations and set together. The data are presented as the fold change compared with the control group (vehicle-treated non-stimulated cells) as the mean ± SEM of 3–5 independent experiments. The results were evaluated using one-way analysis of variance (ANOVA) followed by Bonferroni's *post hoc* test to assess differences between the treatment groups. Significant differences in comparison with the control group (vehicle-treated non-stimulated cells) are indicated by ^*^P < 0.05, ^**^P < 0.01, ^***^P < 0.001; differences between LPS-treated and BoNT/A- and/or MC-treated cells are indicated by ^##^*P* < 0.01.

Additionally, the protein levels of IL-18 were significantly increased after LPS treatment compared to controls in microglia from 1.0 ± 0.3 to 2.8 ± 0.2 (Figure 4B) and astroglia from 1.0 ± 0.1 to 2.9 ± 0.1 (Figure 4F). BoNT/A treatment potentiated the LPS-induced expression levels of IL-18 protein in microglia from 2.8 ± 0.2 to 1.3 ± 0.2 (Figure 4B) but not in astroglia (Figure 4F). Moreover, minocycline potentiated the LPS-induced expression levels of IL-18 in both microglia (from 2.8 ± 0.2 to 1.4 ± 0.1, Figure 5B) and astroglia (from 2.9 ± 0.1 to 2.3 ± 0.2, Figure 4F). The combination treatment significantly reduced IL-18 levels after LPS treatment in microglia from 2.8 ± 0.2 to 1.7 ± 0.5 (Figure 4B) but did not change the effect of LPS on the IL-18 levels in astroglia (from 2.9 ± 0.1 to 2.4 ± 0.2, Figure 4F).

The IL-6 protein levels were significantly increased after LPS treatment compared to controls in microglia from 1.0 ± 0.1 to 4.0 ± 0.2 (Figure 4C) and astroglia from 1.0 ± 0.1 to 3.0 ± 0.2 (Figure 4F). BoNT/A treatment potentiated the LPS-induced expression levels of IL-6 protein in microglia from 4.0 ± 0.2 to 3.1 ± 0.1 (Figure 4C) but not in astroglia (Figure 4G). Moreover, minocycline and the combination treatment potentiated the LPS-induced expression levels of IL-6 in both microglia from 4.0 ± 0.2 to 2.4 ± 0.1 and to 2.3 ± 0.1, respectively (Figure 4C), and astroglia from 3.0 ± 0.2 to 1.6 ± 0.1 and to 2.5 ± 0.1, respectively (Figure 4G).

The levels of NOS2 protein were significantly increased after LPS treatment compared to controls in microglia from 1.0 ± 0.3 to 103.2 ± 6.1 (Figure 4D) and astroglia from 1.0 ± 0.1 to 135.2 ± 9.6 (Figure 4H). BoNT/A treatment decreased the LPS-induced expression levels of NOS2 protein in microglia from 103.2 ± 5.1 to 54.3 ± 4.0 (Figure 4D) but not in astroglia (Figure 4H). Moreover, minocycline and the combination treatment potentiated the LPS-induced expression levels of NOS2 in both microglia from 103.2 ± 5.1 to 39.8 ± 4.0 and from 103.2 ± 5.1 to 73.9 ± 7.0, respectively (Figure 4D), and astroglia from 135.2 ± 9.6 to 108.5 ± 4.0 and from 135.2 ± 9.6 to 96.2 ± 2.9, respectively (Figure 4H).

### The influence of BoNT/A and minocycline on anti-inflammatory factors in vehicle- and LPS-treated microglial and astroglial cells

The mRNA levels of anti-inflammatory factors were significantly decreased (*IL-1RA* and *IL-18BP*) or increased (*IL-10*) after LPS treatment compared to controls in microglial cell cultures. In the astroglial cultures, the LPS-stimulated cells exhibited strong expression of all anti-inflammatory factors. BoNT/A and minocycline treatments did not influence the mRNA levels of *IL-1RA, IL-18BP, IL-10* in any of glial cultures (please see Supplementary Material).

The IL-1RA protein levels were decreased in the microglia from 1.0 ± 0.1 to 0.4 ± 0.1 (Figure 5A) and in the astroglia from 1.0 ± 0.0 to 0.7 ± 0.1 (Figure 5D) in LPS-stimulated cells compared with that of non-stimulated cells. There were no changes in the expression levels of IL-1RA in vehicle-treated cells and LPS-stimulated cells treated with BoNT/A and minocycline alone or the combination treatments in any of glial cultures (Figures 5A,D).

**Figure 6:**
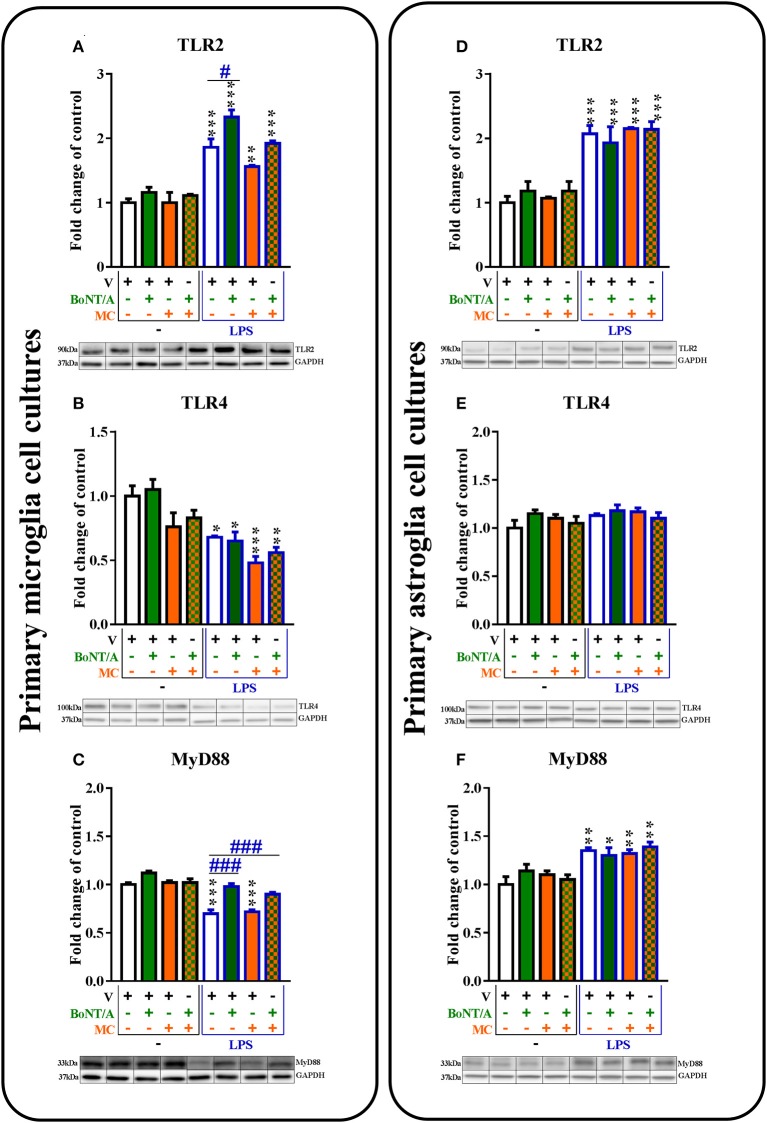
**The influence of BoNT/A and/or minocycline (MC) on TLR2 (A,D)**, TLR4 **(B,E)**, and MyD88 **(C,F)** protein levels in vehicle- and LPS-treated primary microglial **(A–D)** and astroglial **(E–H)** cell cultures. Microglial and astroglial cells were treated with BoNT/A [0.1 nM] and/or minocycline [20 μM] for 30 min and then with LPS [100 ng/mL] for 24 h **(A–H)**. The representative bands are shown below each column of the respective group on the graph and come from the same membrane photo. Samples from different groups were not next to each other so were cut from different locations and set together. The data are presented as the fold change compared with the control group (vehicle-treated non-stimulated cells) as the mean ± SEM of 3–5 independent experiments. The results were evaluated using one-way analysis of variance (ANOVA) followed by Bonferroni's *post hoc* test to assess differences between the treatment groups. Significant differences in comparison with the control group (vehicle-treated non-stimulated cells) are indicated by ^*^P < 0.05, ^**^P < 0.01, ^***^P < 0.001; differences between LPS-treated and BoNT/A- and/or MC-treated cells are indicated by ^#^*P* < 0.05, ^###^*P* < 0.001.

The protein levels of IL-18BP were significantly decreased in microglia from 1.0 ± 0.1 to 0.5 ± 0.1 (Figure 6B) and significantly increased in astroglia from 1.0 ± 0.0 to 1.27 ± 0.0 (Figure 5E) after LPS treatment compared to controls. There were no changes in the expression levels of IL-18BP in LPS-stimulated cells pretreated with BoNT/A and minocycline alone or in combination in any of glial cultures (Figures 5B,E).

The protein levels of IL-10 were significantly increased in microglia from 1.0 ± 0.1 to 2.13 ± 0.2 (Figure 5C) and in astroglia from 1.0 ± 0.1 to 1.2 ± 0.0 (Figure 5F) after LPS treatment compared to controls. BoNT/A decreased IL-10 protein levels after LPS stimulation alone from 2.13 ± 0.2 to 1.3 ± 0.4 (Figure 5C) and/or in the presence of minocycline from 2.13 ± 0.2 to 1.4 ± 0.1 (Figure 5C) in microglia. However, there were no changes in the expression levels of IL-10 in vehicle-treated cells and LPS-stimulated cells with BoNT/A and minocycline alone or in combination in astroglia (Figure 5F).

### The influence of BoNT/A and minocycline on TLR2, TLR4, and their adapter protein MyD88 in vehicle- and LPS-treated microglial and astroglial cells

The levels of *TLR2* mRNA were significantly decreased in microglia and astroglia after LPS treatment compared to controls. The mRNA levels of *TLR4* were decreased in both glial cell cultures in LPS-stimulated cells. The BoNT/A and minocycline treatments did not affect the mRNA levels of *TLR2* and *TLR4* in any of glial cultures (please see Supplementary Material).

The TLR2 protein levels were significantly increased after LPS treatment compared to vehicle-treated non-stimulated cells in microglial (from 1.0 ± 0.1 to 1.9 ± 0.1) and astroglial (from 1.0 ± 0.1 to 2.0 ± 0.1) cell cultures (Figures 6A,D). BoNT/A significantly increased TLR2 protein levels in LPS-stimulated cells compared with that of vehicle-treated LPS-stimulated cells from 1.9 ± 0.1 to 1.1 ± 0.0 in microglia (Figure 6A) but not in astroglia (Figure 6D). There were no changes in the expression levels of TLR2 in vehicle-treated cells and LPS-stimulated cells with minocycline alone or in the presence of BoNT/A in any of glial cultures (Figures 6A,D).

The TLR4 protein levels were significantly decreased after LPS treatment compared to controls in microglial cell cultures from 1.0 ± 0.1 to 0.7 ± 0.0 (Figure 6B) but not in astroglial cell cultures (Figure 6E). There were no changes in the expression levels of TLR4 in vehicle-treated cells and LPS-stimulated cells treated with BoNT/A and minocycline alone or the combination treatments in any of glial cultures (Figures 6B,E).

The MyD88 protein levels were significantly decreased in microglial cell cultures from 1.0 ± 0.0 to 0.7 ± 0.0 (Figure 6C) and significantly increased in astroglial cell cultures from 1.0 ± 0.1 to 1.35 ± 0.0 (Figure 6C) after LPS treatment compared to controls. BoNT/A significantly increased the levels of MyD88 protein in LPS-stimulated cells compared with that of vehicle-treated LPS-stimulated cells from 0.7 ± 0.0 to 1.0 ± 0.0 in microglia (Figure 6C) but not in astroglia (Figure 6F). There were no changes in the expression levels of MyD88 in vehicle-treated cells and LPS-stimulated cells with minocycline alone in any of glial cultures (Figures 6C,F). The combination treatments increased only the MyD88 protein levels after LPS stimulation in the microglial cell cultures from 0.7 ± 0.0 to 0.9 ± 0.0 (Figure 6C).

## Discussion

The results demonstrate the effects of BoNT/A on glial cells. We have revealed that BoNT/A inhibits the expression of pro-inflammatory factors through the modulation of NF-κB, p38, and ERK1/2, and increases the expression of TLR2 and its adaptor protein, MyD88, in microglial cells. However, we did not observe any effects of BoNT/A on those factors in astroglial cell cultures. In contrast, minocycline, the inhibitor of microglial activation, also directly affects astrocytes. Interestingly, minocycline decreases not only the expression of well-known molecular targets, such as p38 and MMP9, but also down-regulates other signaling cascades and many pro-inflammatory factors in both primary glial cultures. Glial cells, mainly microglia and astrocytes, are a source of many inflammatory mediators, (DeLeo and Yezierski, 2001; Mika et al., 2013; Rojewska et al., 2014; Malek et al., 2015; Piotrowska et al., 2016). Presently, it is being suggested that the promotion of glial cell activity which leads to reduced neuroinflammation may be a target of effective therapies for CNS pathologies, such as neuropathic pain.

BoNT/A inhibits the development of neuropathic pain (Luvisetto et al., 2007; Mika et al., 2011; Zychowska et al., 2016) not only by inhibition of neuronal activity but also by affecting glial cell activation (Mika et al., 2011; Vacca et al., 2013; Zychowska et al., 2016). However, the mechanisms of the influence of BoNT/A on glial cells have not yet been elucidated. Recently, a few studies have revealed a beneficial role of BoNT/A on cell viability (Bandala et al., 2015). In 2015, Kim et al. demonstrated that BoNT/A suppressed LPS-induced NO and TNFα production in RAW264.7 macrophages by blocking the activation of JNK, ERK, and p38 MAPK (Kim et al., 2015).

The well-defined molecular targets of BoNT/A action are SNARE proteins, in particular SNAP-25. Our present *in vitro* primary cell culture studies have revealed that microglia possess both mRNA and protein for SNAP-23 but not for SNAP-25, while astrocytes express both. Our results are in agreement with the studies of Hepp et al. (1999) who showed that SNAP-25 is replaced by SNAP-23, a homolog present in microglia and oligodendrocytes. SNAP-23 is structurally and functionally similar to SNAP-25. In the study by Parpura et al. (1995), the expression of some of the SNARE protein complexes, but not SNAP-25, was identified. However, the authors only analyzed post-nuclear astrocytic cell membrane extract, not the whole lysates as we did, and this may explain some of the discrepancies between these two studies. In 2012, Marinelli et al. showed that BoNT/A exerts analgesic effects on neuropathic pain through the cleavage of SNAP-25 in spinal astrocytes and did not observe co-localization with microglia (Marinelli et al., 2012). We showed that BoNT/A decreased microglial SNAP-23 and astroglial SNAP-23 and -25 expression levels after LPS treatment. Interestingly, LPS treatment induced an increase in the levels of SNAP-23 and -25 in astrocytes but diminished the levels of SNAP-23 in microglia. There are no data in the literature that indicate whether minocycline can regulate SNAP-23 or -25 expression, and whether minocycline has an impact on the effect of BoNT/A. Our results demonstrate that minocycline has no effect on the levels of any of the proteins in glial cultures.

In 2015, Kim et al. identified altered biological processes induced by BoNT/A treatment in RAW264.7 macrophages (Kim et al., 2015). It was also shown that BoNT/A especially modulates processes related to signal transduction and immunity/defense, and a further analysis identified cytokine–cytokine receptor interactions and TLRs and MAPKs as the main targets of BoNT/A. There is a paucity of information concerning the effects of BoNT/A on microglial cells, which are known as the resident tissue macrophages in the CNS (Ginhoux et al., 2010). Activation of the microglia leads to the induction of intracellular pathways and the release of many neuromodulatory compounds, which may participate in the alteration of the functions of microglia and neighboring cells (Popiolek-Barczyk and Mika, 2016). Some of the crucial participants of these intracellular signaling pathways, which were also shown to modulate the nociceptive response, are mitogen-activated protein kinases (MAPKs), mainly p38 and ERK1/2. Data from animal studies revealed that the inhibition of both members of the MAPK family leads to the diminishing of neuropathy symptoms, down-regulation of pro-inflammatory factors and enhancement of opioid analgesia (Jin et al., 2003; Tsuda et al., 2004; Mika et al., 2009; Popiolek-Barczyk et al., 2014a,b; Rojewska et al., 2014, 2015; Piotrowska et al., 2016). The results of our present study have shown that BoNT/A diminished phosphorylation of p38 and ERK1/2 after a 1 h incubation with LPS in microglial cells. Our data are in agreement with a previous report derived from macrophage studies, where BoNT/A diminished the phosphorylation of MAPKs (Kim et al., 2015). In our present study, we also determined that minocycline, strongly diminishes the phosphorylation of p38 and ERK1/2. These data are in agreement with the results of a previous study demonstrating that minocycline inhibits the activation of both MAPK family members (Nikodemova et al., 2006). A growing number of studies indicate an essential role for the NF-κB pathway in both nociception and microglial cell activation (Ma and Bisby, 1998; Meunier et al., 2007; Miyoshi et al., 2008; Popiolek-Barczyk et al., 2014b, 2015; Piotrowska et al., 2016). We have shown that the inhibition of NF-κB with a potent inhibitor, parthenolide, not only diminished the symptoms of neuropathy but also potentiated morphine analgesia and reduced the levels of pro-inflammatory factors produced by microglia (IL-1β, IL-18, NOS2) (Popiolek-Barczyk et al., 2014b, 2015). In the present set of experiments, we revealed that BoNT/A diminished NF-κB activation after LPS stimulation in primary microglial cells.

In 2006, Piao et al. reported that the molecular mechanism of action of minocycline consists of the inhibition of p38 in microglia (Piao et al., 2006), while in 2013 Niimi et al. demonstrated that minocycline also inhibits the activity of metalloproteinase 9 (MMP9) (Niimi et al., 2013). In our previous *in vivo* studies, we also confirmed that minocycline prevents CCI-induced microglial cell activation, which was correlated with the decrease of MMP9 protein levels in the spinal cords of rats (Rojewska et al., 2014). Here, we are reporting that minocycline diminishes MMP9 levels in microglia, while the effects of BoNT/A are not significant.

It is already known that BoNT/A injection restores the neuro-immune balance in a CCI model (Zychowska et al., 2016), and our *in vitro* studies with glial cell cultures revealed the inhibitory action of BoNT/A on the intracellular pathways followed by the down-regulation of pro-inflammatory IL-1β, IL-18, IL-6, NOS2. Additionally, minocycline attenuated spinal IL-1β, IL-18, IL-6, and NOS2 (Makuch et al., 2013; Rojewska et al., 2014) of CCI-exposed animals, what is supported by our present study. Similarly, in 2013, Kobayashi et al. indicated that minocycline selectively inhibits IL-1β in microglia and additionally showed a reduction of TNF-α and IFNγ (Kobayashi et al., 2013). Here, we are reporting that minocycline also reduces IL-6, IL-18, NOS2 in LPS-stimulated cells. In the aforementioned work, the authors did not analyze the effects of minocycline on anti-inflammatory factors after LPS treatment. We have proven that minocycline reduces the anti-inflammatory IL-10, however, has no effect on IL-18BP or IL-1RA.

In our present study, we revealed that BoNT/A does not affect the activation of MAPKs, p38, and ERK1/2, or NF-κB in LPS-stimulated astroglial cell cultures. Simultaneously, we did not observe any changes in the mRNA or protein levels of deleterious factors, such as MMP9, IL-1β, IL-18, NOS2. TLRs and their adaptors initiate the activation of NF-κB and MAPK, which is required for the production of inflammatory cytokines (Kawai and Akira, 2010). In contrast, we have noticed that minocycline significantly diminishes the mRNA and protein levels of MMP9 and the phosphorylated forms of p38, ERK1/2, as well as NF-κB and related pro-inflammatory factors (IL-1β, IL-18, IL-6, and NOS2).

Among the various receptors expressed by microglia, the TLR family, especially subtypes 2 and 4, represents a possible link between microglial activation and nerve injury, and plays a crucial role in the development of neuropathic pain symptoms (Lehnardt et al., 2003; Tanga et al., 2005; Kim et al., 2007; Jurga et al., 2016). The TLR family plays a fundamental role in a pathogen recognition and the activation of innate immunity and leads to the induction of direct antimicrobial pathways, expression of co-stimulatory molecules, and release of cytokines via NF-κB and/or MAPK signaling. They can also recognize pathogen-associated molecular patterns (PAMPs), which are expressed on infectious agents (Medzhitov et al., 1997; Borrello et al., 2011), and danger-associated molecular patterns (DAMPs), which are products of nerve injury (Liu et al., 2012), and mediate the production of cytokines. It has been demonstrated that genetically altered mice lacking TLR2 or TLR4 show markedly decreased microglial activation, with a parallel reduction in neuropathic pain symptoms (Tanga et al., 2005; Kim et al., 2007). Our results confirm the roles of the TLR2 and TLR4 receptors in nociceptive transmission (Jurga et al., 2016). In the CNS, TLR2, and TLR4 are predominantly expressed on glial cells, with the greatest importance in the context of neuropathy on microglia (Kim et al., 2007; Miyake, 2007). Our *in vitro* results have revealed a decrease in the mRNA and protein levels of TLR4 in microglia, but not in astroglia, after a 24-h stimulation period with LPS, and neither BoNT/A nor minocycline had an effect on its expression. Additionally, studies using macrophages showed that BoNT/A is sensed by TLR2 but not by TLR4 (Kim et al., 2015). Similarly, minocycline did not affect the levels of TLR2. In turn, BoNT/A significantly increased the mRNA and protein levels of TLR2 in LPS-stimulated microglia but not in astrocytes. It has been shown that TLR4 activation is mediated by dimerization of adapter proteins such as MyD88 or TRIF, but TLR2 uses only MyD88 (Kigerl et al., 2014). Our *in vitro* results have shown that BoNT/A significantly rescues LPS-reduced levels of MyD88 protein in microglia but does not affect its expression in astrocytes. Interestingly, the latest reports indicate an interaction between TLR signaling and SNARE proteins. MyD88-dependent TLR signaling is involved in the phosphorylation of SNAP-23 present on the phagosome in dendritic cells. Phospho-SNAP-23 stabilizes SNARE complexes, what leads to a fusion with the endosomal recycling compartment and ultimately cross-presentation (Nair-Gupta et al., 2014). A similar interaction may also occur in microglial cells, because they act as the first and main form of active immune defense in the CNS and become fully competent antigen-presenting cells (Beauvillain et al., 2008). Thus, it appears that the microglial TLR-MyD88-NF-κB pathway contributes to the reduction of SNAP-23. Several reports suggest that microglia are characterized by increased expression levels of TLRs and a stronger response to LPS compared to astrocytes (Holm et al., 2012; Facci et al., 2014). Moreover, Holm et al. (2012) revealed that the response of astrocytes to TLR2 agonists is completely dependent on the presence of functional microglial TLR4. In addition, the activation of TLR4 by LPS induces a synthesis of the closely related TLR2 (Lin et al., 2000). In our studies, we did not observe any changes in the expression levels of the analyzed factors with the exception of SNAP-23 and -25 after BoNT/A treatment in non-stimulated and LPS-activated primary astroglial cell cultures. We hypothesized that TLR2 is another molecular target for BoNT/A. It appears that the weakened effect of BoNT/A on astrocytes and their TLR2 signaling-dependent immune response can be explained by the results obtained by Holm et al. (2012) where the authors proved that this glial crosstalk and the presence of the TLR4 receptors on microglia are required to enhance the effects of BoNT/A. However, this issue needs to be addressed in future studies.

## Conclusions

**Figure 7:**
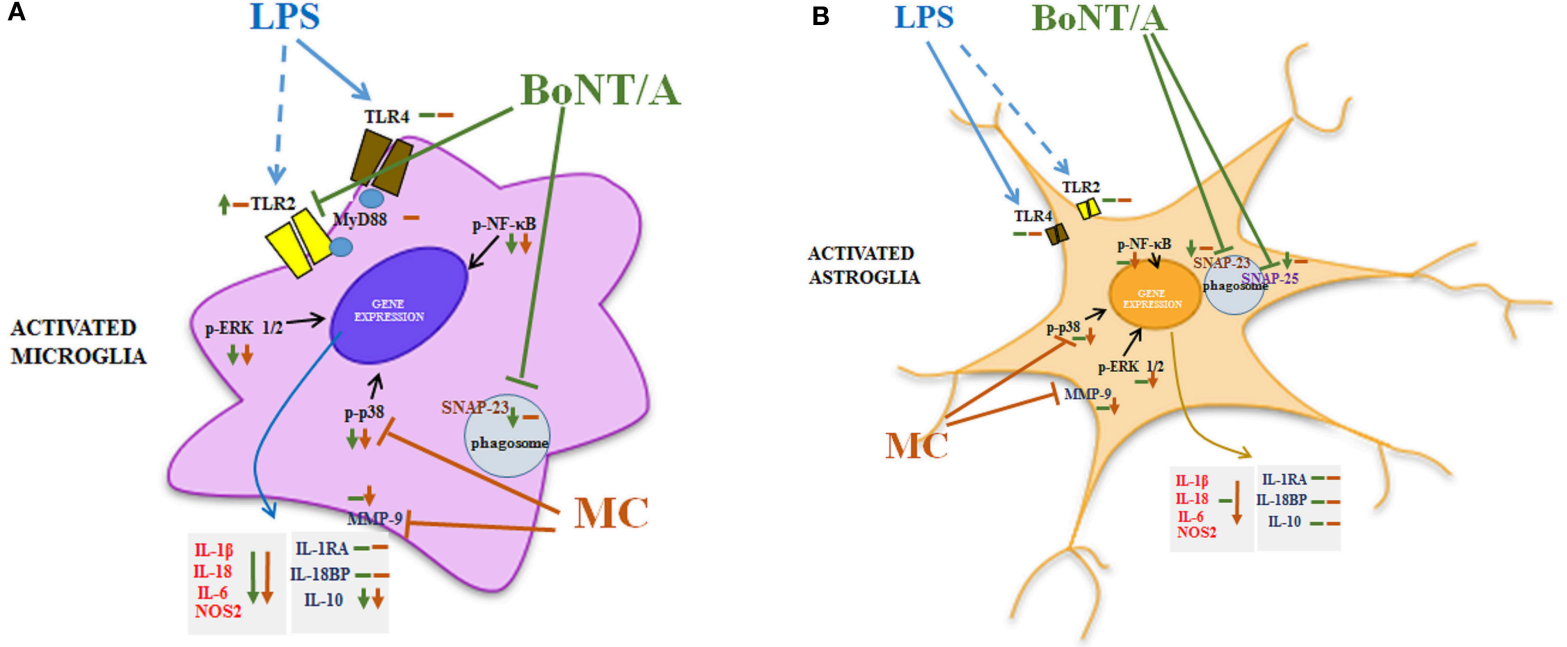
**The hypothetical mechanism of action of BoNT/A is achieved through the modulation of TLR2 and SNARE proteins, and minocycline occurs through the modulation of p38 and MMP9 in glial cells**. Our studies revealed that BoNT/A significantly diminishes LPS-induced phosphorylation of p38, ERK1/2, and NF-κB in microglial cells in primary cultures. Moreover, BoNT/A treatment leads to a reduction in the release of pro-inflammatory factors, such as IL-1β, IL-18, IL-6, and anti-inflammatory IL-10. We did not observe any effects of BoNT/A on primary cultures of astrocytes. Minocycline also inhibits LPS-induced phosphorylation of p38, ERK1/2 and NF-κB in microglial primary cultures. Moreover, we obtained similar effects in astrocyte cultures. The results of our studies are in agreement with data obtained by others, suggesting that minocycline diminishes both pro-inflammatory (IL-1β, IL-18, IL-6) and anti-inflammatory (IL-10) factors in microglia, as well as astrocytes, as a result of a direct action on p38 and MMP9 (Piao et al., 2006; Niimi et al., 2013). The activation of p38 is correlated with the regulation of synthesis of interleukins, and the role of MMP9 is to transform an inactive form of IL-1β (pro-IL-1β) into active IL-1β (Beyaert et al., 1996; Kawasaki et al., 2008). As previously shown, the activation of TLR4 by LPS leads to an increased expression of TLR2 and induction of its activation (Lin et al., 2000). However, because of the lower expression levels of TLR4 in astrocytes, we did not observe signal amplification between TLR4-TLR2. In a previous study, it was suggested that TLR2 receptors are molecular targets of BoNT/A (Kim et al., 2015). In primary microglial cell cultures, we observed a significant reduction in TLR4 expression after LPS stimulation. This TLR4 reduction is correlated with a receptor activation and internalization, and it is followed by an increased expression of TLR2. Additionally, BoNT/A, but not minocycline, potentiates the level of TLR2 after 24 h of LPS treatment. This effect was also observed by Kim et al. (2015) in a macrophage cell line. It is well documented that the activation of TLRs initiates intracellular cascades activation, mainly NF-κB and MAPKs (Kawai and Akira, 2010). Moreover, in microglial cell cultures we observed a simultaneous increase in the levels of SNAP-23 and TLR2 after 24 h of LPS treatment. In 2014, Nair-Gupta et al. revealed that the TLR-MyD88-NF-κB pathway is involved in the phosphorylation of SNAP-23 (Nair-Gupta et al., 2014). The results of our data from primary glial cell cultures showed that BoNT/A diminishes microglial SNAP-23 and astrocytic SNAP-23 and -25. We also revealed that minocycline does not influence the levels of SNARE proteins. As it was suggested by Holm et al. (2012), a complete activation of TLR2 in astrocytes requires the presence of the microglial TLR4 receptor. Therefore, we hypothesized that glial crosstalk may explain the lack of effect of BoNT/A on astrocytes. At the same time, we suggest that the molecular target of BoNT/A is TLR2. Abbreviations: SNAP, synaptosomal-associated protein; TLR, Toll-like receptor; MyD, myeloid differentiation primary response gene; ERK1/2, extracellular signal-regulated kinase 1/2; NF-κB, nuclear factor-κ B; NOS2, inducible nitric oxide synthase; IL, interleukin; MMP, matrix metallopeptidase; LPS, lipopolysaccharide, BoNT/A, botulinum toxin serotype A; MC, minocycline.

Our pioneering research indicates that BoNT/A exerts its anti-inflammatory action by inhibiting NF-κB, p38, and ERK1/2 activation in microglial cells and directly interacts with TLR2. Additionally, BoNT/A only appears to have slight, if any, effect on astrocytes. In contrast, minocycline not only affects microglia but also influences astrocytes. The modulation of p38 and MMP9, the molecular targets of minocycline, seems to be partially responsible for its anti-inflammatory action, which is not the case for BoNT/A. Thus, it seems that the main target of BoNT/A is TLR2. Minocycline and BoNT/A, despite their different molecular targets, modulate the same intracellular pathways and achieve the same final results in primary microglial cell cultures. These findings provide a likely explanation for our previous *in vivo* experiments in a neuropathic pain model, where we observed that microglial inhibition using minocycline only slightly enhanced the analgesic effects of BoNT/A. In addition, on the basis of the results obtained and the current state of knowledge we have created the hypothesis that the full activation of TLR2 in astrocytes requires the presence of functional TLR4 in microglial cells, which emphasizes the significant interaction between both types of cells. This, however, requires further studies (Figure 7).

## Author contributions

AP, KP, FP, and JM have substantial contributions to the conception, design of the study, analysis and interpretation of data for the work; Final approval of the version to be published; Agreement to be accountable for all aspects of the work in ensuring that questions related to the accuracy or integrity of any part of the work are appropriately investigated and resolved.

## Funding

This work was supported by the National Science Centre, Poland—grants HARMONIA 5 2013/10/M/NZ4/00261, OPUS 11 2016/21/B/NZ4/00128 & PRELUDIUM 2012/07/N/NZ3/00379 and statutory funds of the Institute of Pharmacology Polish Academy of Sciences. A. Piotrowska is a Ph.D. student funded by a scholarship from the National Centre of Scientific Leading sponsored by the Ministry of Science and Higher Education, Republic of Poland. The English was corrected by American Journal Experts (certificate no. 821C-AC85-1F23-57DC-FDC5).

### Conflict of interest statement

The authors declare that the research was conducted in the absence of any commercial or financial relationships that could be construed as a potential conflict of interest.
