# Supplementary material for: Comparison of the Expression Changes after Botulinum Toxin Type A and Minocycline Administration in Lipopolysaccharide-Stimulated Rat Microglial and Astroglial Cultures
Source: Front Cell Infect Microbiol. 2017 Apr 26;7:141. doi: 10.3389/fcimb.2017.00141 (PMC5405066; doi:10.3389/fcimb.2017.00141)
Supplement: Supplementary file 1 [file DataSheet1.docx]

**SUPPLEMENTARY MATERIALS**

**SUPPLEMENTARY 1 – RT-PCR ANALYSIS**


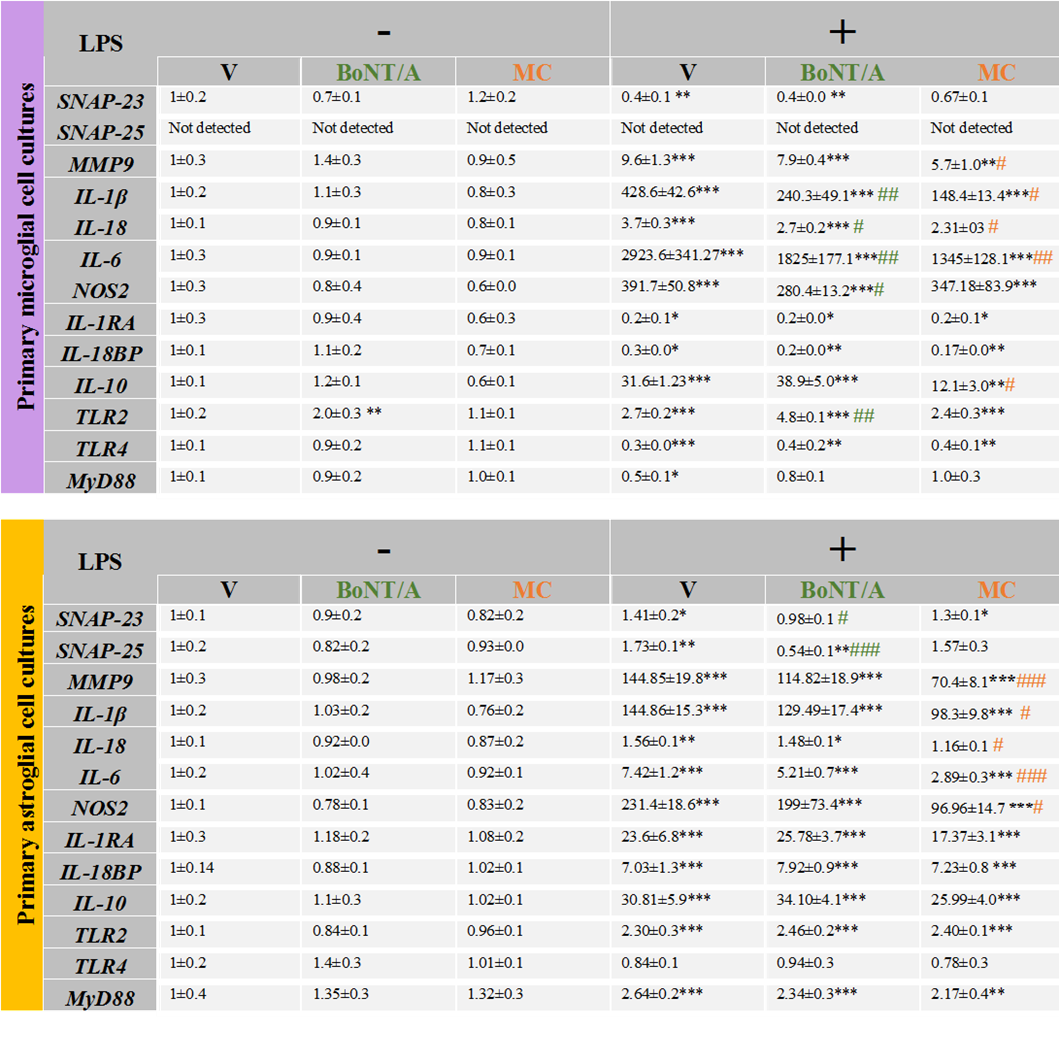


**Table 1.** The influence of BoNT/A or minocycline (MC) on *SNAP-23*, *SNAP-25, MMP9, IL-1β*, *IL-18, IL-6*, *NOS2*, *IL-1RA*, *IL-18BP*, *IL-10*, *TLR2*, *TLR4*, and *MyD88* mRNA levels in vehicle- and LPS-treated primary microglial (**Purple table**) and astroglial (**Yellow table**) cell cultures. Microglial and astroglial cells were treated with BoNT/A [0.1 nM] and minocycline [20 µM] for 30 min and then with LPS [100 ng/mL] for 24 h. The data are presented as the fold change compared with the control group (vehicle-treated non-stimulated cells) as the mean ± SEM of 3-5 independent experiments. The results were evaluated using one-way analysis of variance (ANOVA) followed by Bonferroni’s *post hoc* test to assess differences between the treatment groups. Significant differences in comparison with the control group (vehicle-treated non-stimulated cells) are indicated by **P*<0.05, ***P*<0.01, ****P*<0.001; differences between LPS-treated and BoNT/A- or MC-treated cells are indicated by ^#^*P*<0.05, ^##^*P*<0.01, ^###^*P*<0.001.

**RESULTS**

***The influence of BoNT/A and minocycline on SNARE, MMP9, pro- and anti-inflammatory factors,TLR2, TLR4 and Myd88 mRNA levels in vehicle- and LPS-treated microglial and astroglial cells.***

SNAP-23 mRNA (Table 1A, B) was decreased in microglia and increased in astroglia after LPS stimulation. The mRNA levels of MMP9 were strongly increased after LPS stimulation, and these elevated levels were reduced by minocycline but not BoNT/A in both glial cell types (Table 1A, B). The mRNA levels of pro-inflammatory factors (IL-1β, IL-18, IL-6 and NOS2) were significantly increased after LPS treatment compared to controls in the microglial and astroglial cell cultures (Table 1A, B). BoNT/A treatment decreased the mRNA levels of those factors in microglial (Table 1A) but not astroglial (Table 1B) cell cultures. Minocycline decreased the mRNA levels of those factors in both cell cultures (Table 1A, B). The mRNA levels of anti-inflammatory factors were significantly decreased (IL-1RA and IL-18BP) or increased (IL-10) after LPS treatment compared to controls in microglial cell cultures (Table 1A). In the astroglial cultures, the LPS-stimulated cells exhibited strong expression of all anti-inflammatory factors (Table 1B). BoNT/A and minocycline treatments did not influence the mRNA levels of IL-1RA, IL-18BP, IL-10 in both glial cultures (Table 1A, B). The levels of *TLR2* mRNA were significantly decreased in microglia and astroglia after LPS treatment compared to controls (Table 1A, B). The mRNA levels of *TLR4* were decreased in both glial cell cultures in LPS-stimulated cells (Table 1A, B). The BoNT/A and minocycline treatments did not affect the mRNA levels of *TLR2* and *TLR4* in both glial cultures (Table 1A, B).

**MATERIALS AND METHODS**

**Analysis of gene expression (RT-qPCR)**

Cells were collected from the cerebral cortex at 24 h after LPS stimulation. Total RNA was extracted according to the method described by (Chomczynski and Sacchi, 1987) using TRIzol reagent (Invitrogen). A NanoDrop ND-1000 spectrometer (NanoDrop Technologies, Wilmington, USA) was used to measure the RNA concentration in all probes. Reverse transcription was performed at 37°C for 60 min using Omniscript reverse transcriptase (Qiagen Inc., Hilden, Germany) with 500 μg of total RNA from the cells. The reaction was performed in the presence of an RNAse inhibitor (rRNasin, Promega, Mannheim, Germany) and an oligo (dT16) primer (Qiagen Inc., Hilden, Germany). The resulting cDNA was diluted 1:10 with H2O, and approximately 50 ng of cDNA from each individual animal was used for each quantitative real-time PCR (RT-qPCR) reaction. The RT-qPCR was performed using Assay-On-Demand TaqMan probes according to the manufacturer’s protocol (Applied Biosystems, Foster City, CA, USA) and run in an iCycler device (Bio-Rad, Hercules, Warsaw, Poland). A standard dilution curve was used to determine the amplification efficiency for each assay. The following TaqMan primers and probes were used: Rn01527838_g1 (HPRT, hypoxanthine-guanine phosphoribosyltransferase); Rn00580432_m1 (IL-1beta, interleukin-1 beta), Rn02586400_m1 (IL-1RA, interleukin-1 receptor antagonist), Rn01422083_m1 (IL-18, interleukin-18), Rn00584495_q1 (IL-18BP, interleukin-18 binding protein), Rn00561420_m1 (IL-6, interleukin-6), Rn00563409_m1 (IL-10, interleukin-10), Rn00561646_m1 (NOS2, inducible nitric oxide synthase), Rn03035106_s1 (SNAP-23, synaptosomal-associated protein 23), Rn00578534_m1 (SNAP-25, synaptosomal-associated protein 25), Rn02133647_s1 (TLR2, Toll-like receptor 2), Rn00569848_m1 (TLR4, Toll-like receptor 2), and Rn01640049_m1 (MyD88, myeloid differentiation primary response gene 88). A standard dilution curve was used to determine the amplification efficiency for each assay (between 1.7 and 2). The cycle threshold values were calculated automatically by CFX Manager v.2.1 (Bio-Rad, Warsaw, Poland) software according to the default parameters. RNA content was calculated as 2 - (threshold cycle). HPRT transcript level was not significantly changed in the LPS-treated cells (Piotrowska et al.,2016) and therefore served as an adequate housekeeping gene.

**SUPPLEMENTARY 2 - LPS STIMULATION**

LPS from Sigma-Aldrich (Lipopolysaccharides from Escherichia coli O111:B4 γ-irradiated, BioXtra; product specification: <http://www.sigmaaldrich.com/catalog/product/sigma/l4391?lang=pl&region=PL>) was used. It is a premium product and suitable for cell culture. This LPS was already used in our earlier studies for few years (Piotrowska et al., 2016a,b; Rojewska et al., 2016; Popiolek-Barczyk et al., 2015; Rojewska et al., 2014; Popiolek-Barczyk et al., 2014). Therefore for reproducibility of our data and the possibility of comparison with earlier studies, the same LPS was used. This particular Sigma-Aldrich product was used in the research of many scientists (at the Company web site there is 82 references where this product was used, e.g. Zhang et al., 2015; Hong et al., 2015; Verheijden et al. 2015; Yu et al., 2014; Enstrom et al., 2010). In 2010 Douville et al. has shown that used by us LPS purity was validated by the finding that pretreatment with TLR4 blocking antibodies (anti-CD284/TLR4), but not IgG2a isotype control antibodies, blocks the capacity of LPS to stimulate cytokine production demonstrate its specificity for TLR4 (Douville et al. Figure S1 https://www.ncbi.nlm.nih.gov/pmc/articles/PMC2919413/bin/pone.0012087.s001.tif).

Primary microglial and astrocyte cell cultures were LPS-treated [100 ng/mL] (lipopolysaccharide from Escherichia coli 0111:B4; Sigma-Aldrich, St. Louis, USA) and incubated for 1 h (for analysis of intracellular pathway activation) and 24 h (for analysis of gene expression, MTT) (Piotrowska et al., 2016; Rojewska et al., 2016; Popiolek-Barczyk et al., 2015). The chosen time points was based on literature data (Zhao et al., 2014; Ellert-Miklaszewska et al., 2013; Kobayashi et al., 2013), as well as our own experience (Piotrowska et al., 2016; Rojewska et al., 2016; Popiolek-Barczyk et al, 2015). The analysis of phosphorylation of intracellular pathways was carried out mainly at the early stages after cell stimulation. Our study show low (not significantly different from un-stimulated cells) activation of intracellular pathways after 24h LPS stimulation (Supplementary Fig. 1). In our present sets of experiments we decided to performed this analysis 1h after LPS stimulation as it was shown in our earlier studies (Piotrowska et al., 2016; Rojewska et al., 2016), as well as by others (Zhao et al., 2014; Ellert-Miklaszewska et al., 2013; Kobayashi et al., 2013). While, the analysis of immunological factors and TLR receptors expression was after 24h, based on literature data (Kobayashi et al., 2013) and our earlier studies (Piotrowska et al., 2016; Popiolek-Barczyk et al, 2015).

**Supplementary Figure 1.** Phosphorylation of p38MAPK and ERK1/2 after 24h LPS stimulation in primary microglial and astroglial cell cultures.

**References**

**Our selected papers**

Piotrowska, A., Kwiatkowski, K., Rojewska, E., Makuch, W., Mika, J. (2016) Maraviroc reduces neuropathic pain through polarization of microglia and astroglia - Evidence from in vivo and in vitro studies. Neuropharmacology. 108:207-19. doi: 10.1016/j.neuropharm.2016.04.024.

Piotrowska, A., Kwiatkowski, K., Rojewska, E., Slusarczyk, J., Makuch, W., Basta-Kaim, A., Przewlocka, B., Mika, J. (2016) Direct and indirect pharmacological modulation of CCL2/CCR2 pathway results in attenuation of neuropathic pain - In vivo and in vitro evidence. J Neuroimmunol. 297:9-19. doi: 10.1016/j.jneuroim.2016.04.017.

Rojewska, E., Piotrowska, A., Makuch, W., Przewlocka, B., Mika J. (2016) Pharmacological kynurenine 3-monooxygenase enzyme inhibition significantly reduces neuropathic pain in a rat model. Neuropharmacology. 102:80-91. doi: 10.1016/j.neuropharm.2015.10.040.

Popiolek-Barczyk, K., Kolosowska, N., Piotrowska, A., Makuch, W., Rojewska, E., Jurga, A.M., Pilat, D., Mika, J. (2015) Parthenolide Relieves Pain and Promotes M2 Microglia/Macrophage Polarization in Rat Model of Neuropathy. Neural Plast. 2015:676473. doi: 10.1155/2015/676473.

**Others selected papers**

Chomczynski, P., and Sacchi, N. (1987). Single-step method of RNA isolation by acid guanidinium thiocyanate-phenol-chloroform extraction. Anal. Biochem. 162, 156–159. doi: 10.1016/0003-2697(87)90021-2

Douville, R.N., Lissitsyn, Y., Hirschfeld, A.F., Becker, A.B., Kozyrskyj, A.L., Liem, J., Bastien, N., Li, Y., Victor, R.E., Sekhon, M., Turvey, S.E,, HayGlass, K.T. (2010) TLR4 Asp299Gly and Thr399Ile polymorphisms: no impact on human immune responsiveness to LPS or respiratory syncytial virus. PLoS One. 10;5(8):e12087. doi: 10.1371/journal.pone.0012087.

Ellert-Miklaszewska, A., Dabrowski, M., Lipko, M., Sliwa, M., Maleszewska, M., Kaminska, B. (2013) Molecular definition of the pro-tumorigenic phenotype of glioma-activated microglia. Glia. 61(7):1178-90. doi: 10.1002/glia.22510. Epub 2013 May 7.

Hong, S., Dimitrov, S., Cheng, T., Redwine, L., Pruitt, C., Mills, P.J., Ziegler, M.G., Green, J.M., Shaikh, F., Wilson, K. (2015) Beta-adrenergic receptor mediated inflammation control by monocytes is associated with blood pressure and risk factors for cardiovascular disease. Brain Behav Immun. 50:31-8. doi: 10.1016/j.bbi.2015.08.012.

Kobayashi, K., Imagama, S., Ohgomori, T., Hirano, K., Uchimura, K., Sakamoto, K., *et al.* (2013) Minocycline selectively inhibits M1 polarization of microglia., *Cell Death & Disease*, 4 (3), pp. e525. DOI:10.1038/cddis.2013.54.

Verheijden, S., Beckers, L., Casazza, A., Butovsky, O., Mazzone, M., Baes, M. (2015) Identification of a chronic non-neurodegenerative microglia activation state in a mouse model of peroxisomal β-oxidation deficiency. Glia. 63(9):1606-20. doi: 10.1002/glia.22831.

Yu, S., Nie, Y., Knowles, B., Sakamori, R., Stypulkowski, E., Patel, C., Das, S., Douard, V., Ferraris, R.P., Bonder, E.M., Goldenring, J.R., Ip, Y.T., Gao, N. (2014) TLR sorting by Rab11 endosomes maintains intestinal epithelial-microbial homeostasis. EMBO J.33(17):1882-95. doi: 10.15252/embj.201487888.

Zhang, W.C., Zheng, X.J., Du, L.J., Sun, J.Y., Shen, Z.X., Shi, C., Sun, S., Zhang, Z., Chen, X.Q., Qin, M., Liu, X., Tao, J., Jia, L., Fan, H.Y., Zhou, B., Yu, Y., Ying, H., Hui, L., Liu, X., Yi, X., Liu, X., Zhang, L., Duan, S.Z. (2015). High salt primes a specific activation state of macrophages, M(Na). Cell Res. 25(8):893-910. doi: 10.1038/cr.2015.87.
